# *Inter-body coupling* in electro-quasistatic human body communication: theory and analysis of security and interference properties

**DOI:** 10.1038/s41598-020-79788-9

**Published:** 2021-02-23

**Authors:** Mayukh Nath, Shovan Maity, Shitij Avlani, Scott Weigand, Shreyas Sen

**Affiliations:** 1 School of Electrical and Computer Engineering, Purdue University, West Lafayette, USA; 2 Eli Lilly and Company, Indianapolis, USA

**Keywords:** Electrical and electronic engineering, Biomedical engineering

## Abstract

Radiative communication using electromagnetic fields is the backbone of today’s wirelessly connected world, which implies that the physical signals are available for malicious interceptors to snoop within a 5–10 m distance, also increasing interference and reducing channel capacity. Recently, Electro-quasistatic Human Body Communication (EQS-HBC) was demonstrated which utilizes the human body’s conductive properties to communicate without radiating the signals outside the body. Previous experiments showed that an attack with an antenna was unsuccessful at a distance more than 1 cm from the body surface and 15 cm from an EQS-HBC device. However, since this is a new communication modality, it calls for an investigation of new attack modalities—that can potentially exploit the physics utilized in EQS-HBC to break the system. In this study, we present a novel attack method for EQS-HBC devices, using the body of the attacker itself as a coupling surface and capacitive inter-body coupling between the user and the attacker. We develop theoretical understanding backed by experimental results for inter-body coupling, as a function of distance between the subjects. We utilize this newly developed understanding to design EQS-HBC transmitters that minimizes the attack distance through inter-body coupling, as well as the interference among multiple EQS-HBC users due to inter-body coupling. This understanding will allow us to develop more secure and robust EQS-HBC based body area networks in the future.

## Introduction

*Wireless communication using electromagnetic radiation* has formed the base-bone for today’s ubiquitous connected devices with a possibility of trillions of connected ‘things’—forming the ‘Internet of Things’ (IoT) revolution. A portion of these IoT devices will be on, around or even inside the human body creating a network of intelligent devices - namely the ‘Internet of Body’ (IoB). The distinguishing feature for IoB devices compared to IoT devices is that IoB devices share a common medium - i.e. the body itself^1^.

**Figure 1:**
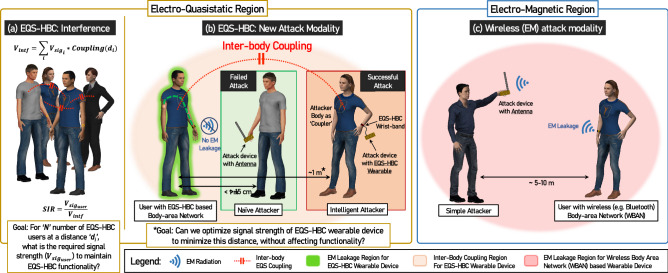
Inter-body coupling in Electro-quasistatic region: (**a**) Interference in received EQS-HBC signal due to inter-body coupling with other users. For multiple EQS-HBC users in close proximity, the received signal is usable only if the interference signal is a few dB lower than the signal. (**b**) While EQS-HBC devices restrict EM leakage within 10 cm of the user’s body, inter-body capacitive coupling can give rise to a new attack modality, where the attacker’s body is used to capacitively couple to the user’s body, and the coupled signal is picked up using an EQS-HBC receiver. (**c**) For devices that do not restrict EM leakage, such as Bluetooth or other WBAN devices, the signal can be picked up by an attacking device with an antenna within 5–10 m of the user. *The human figures were created using the open-source software ‘MakeHuman’*^12^.

Since traditional Body Area Network (BAN) devices operate through radiative communication such as Bluetooth, Med-Radio, WiFi etc, the physical signals are not only available on and around the user’s body, but also broadcast away from the user—making it available for malicious interceptors within 5–10 m distance (Fig. 1c). This brings us to the natural question: can the distinguished feature, i.e. the body as a common medium, be used to improve the security of IoB devices?

Recently, Electro-Quasistatic Human Body Communication (EQS-HBC)^2^ was introduced as a “Physically Secure” way to communicate among IoB devices using the body itself as a ‘wire’^3^. Unlike traditional WBAN devices, frequencies used in EQS-HBC are low ($< 1 \,\hbox {MHz}$)—such that the corresponding wavelength is large with respect to the human body, making the communication electro-quasistatic (EQS) in nature. EQS-HBC, more specifically capacitive EQS-HBC, uses the human body as a forward path in a circuit to transmit signal between a transmitter and a receiver, and completes the communication path through parasitic capacitive coupling formed between the EQS-HBC device’s floating ground and earth’s ground. As the human body—acting as an electrically small antenna—does not radiate well in the EQS frequency regime, it makes the EQS-HBC communication path analogous to a closed loop electrical circuit. As demonstrated by Das et al^2^—since far-field electromagnetic radiation of signal is prevented in EQS-HBC signal, the signal is restricted within 1 cm of the body surface and 15 cm of an EQS-HBC device—making it physically secure.

However, one may raise a question, whether an E-field probe or an antenna is indeed the best way to attack or sniff EQS-HBC communication. We motivate this discussion by considering the fact that EQS-HBC uses the human body as medium, and asking the question: Is there any way two human bodies can couple, making EQS-HBC signals available on a second person’s body? For Radiative communication protocols such as Bluetooth, the signal propagation between a transmitters and receiver is well-understood, and can be estimated well using the Friis Transmission Eq. ^4^:

1 $$\begin{aligned} \frac{P_{Rx}}{P_{Tx}} \propto \left( \frac{\lambda }{d}\right) ^{{n}} \end{aligned}$$

where $P_{Rx}$ and $P_{Tx}$ are received power and transmitted power respectively and the path loss exponent *n* is determined by people and objects present in the signal path. The Friis equation provides a simple outlook on the distance over which the signal from a radiative device can be picked up. For EQS-HBC systems however, a similar understanding is required, especially in the electro-quasistatic region. Literature survey reveals studies that have considered the human body as an antenna before, and these works fall under mainly two categories—one where the Specific Absorption Rate (SAR) of the human body has been investigated over different frequencies^5–7^, and the other, where the interference received by the human body for incident EM waves has been examined^8,9^. Kibret^8,10^ characterized antenna properties of the human body by modelling it as a monopole antenna in the 1–200 MHz range. Li^11^ used the same approach to examine wireless signal transmission between two humans for frequencies 1–90 MHz. Unfortunately these studies do not directly correspond to EQS-HBC inter-body coupling—because firstly, the frequency ranges explored in these works fall out of the low frequency EQS-HBC range ($< 1 \,\hbox {MHz}$) and they deal with radiative communication that cannot be applied to EQS inter-body coupling. Secondly, these studies do not use wearable devices as transmitters and receivers and hence the results from these studies cannot be applied to EQS-HBC devices that use parasitic coupling between its floating ground and the earth’s ground to close the loop of communication. A theory of human inter-body coupling in the context of EQS-HBC—to the author’s best knowledge—has never been developed before. In this paper, we answer the question of a better attack modality of EQS-HBC by developing, for the first time, an understanding of inter-body coupling over a broad frequency range (100 kHz–1 GHz) along with a detailed focus on the EQS region. We show that the human body can function as a capacitor plate in the EQS region and an attack device connected to the attackers body can potentially “sniff” EQS-HBC signals from a further distance, compared to an attack device connected to an antenna (Fig. 1b). Using the developed theory and understandings of the physical principles, we propose an improved EQS-HBC communication design that is tolerant of “Inter-Body Attack” as well as minimizes inter-human interference (Fig. 1a), thus improving channel capacity.

**Figure 2:**
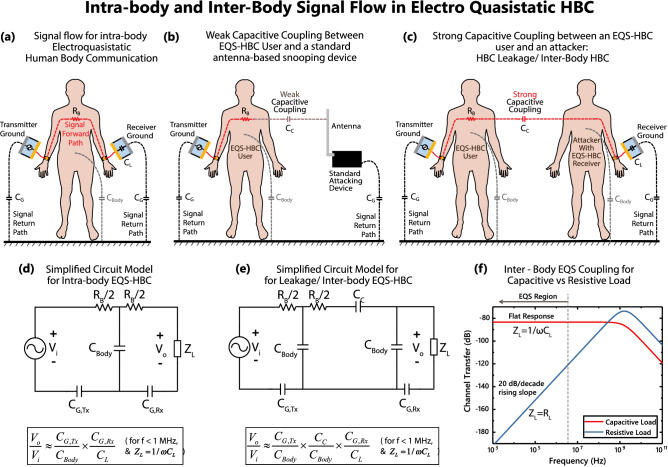
(**a**) Forward and return path for regular intra-body Electro-quasistatic Human Body communication (EQS-HBC). Forward path is formed through the human body, while the return path is formed through parasitic capacitances $C_{G,Tx}$ and $C_{G,Rx}$ with environment. (**b**) Weak Capacitive coupling between an EQS-HBC user and an antenna, ensuring minimal leakage pick-up by that antenna. This implies minimal interference and maximum security towards antenna based devices. (**c**) Strong capacitive coupling, $C_C$ between two human bodies pose the question of inter-body signal leakage for EQS HBC. This can potentially allow the 2nd user, the attacker, to sniff EQS HBC signals from the 1st user. If the 2nd person is just a regular user of HBC, the capacitive coupling can cause interference between the EQS HBC Signals from the two bodies. (**d**) Simplified circuit model for regular intra-body HBC of Fig. 2a, and approximate expression for channel loss. (**e**) Simplified circuit model for inter-body HBC or HBC leakage, from Fig. 2b, and approximate expression for channel loss. The extra term $C_C/C_{Body}$ represents an additional loss from inter-body coupling. (**f**) Comparison of inter-body EQS coupling for capacitive vs Resistive load at the receiver’s end. For capacitive load, the low-frequency region is a flat-band response. For resistive load, the response is a 20 dB/decade rising slope.

### EQS inter-body coupling

In capacitive EQS-HBC, signal electrodes of the transmitting and receiving devices are connected to a human body, while the ground electrodes are left floating. As shown in Fig. 2a, the human body forms the forward path of communication^13^, while the return path is formed by parasitic capacitance between the earth’s ground and the transmitter and receiver ground planes^14^ ($C_{G,Tx}$ and $C_{G,Rx}$ respectively). This parasitic return path is key in capacitive EQS-HBC operation, as low-frequency EQS operation makes the system analogous to an electrical circuit (2d)—where a closed loop must be present between the transmitter and the receiver. The impedance of the return path capacitances are much higher compared to the forward path resistance $R_{B}$ for frequencies $< 1 \,\hbox {MHz}$^14,15^, and when operated in that frequency region, most of the potential drop happens across $C_{G,Tx}$ and $C_{G,Rx}$. The fact that wavelength of signals are much larger than the human body dimensions, leaves the entire human body roughly at the same quasistatic electric potential—letting us incorporate the body as a single point node in the circuit model and introduce the idea of inter-body coupling simply in terms of a lumped version of a distributed coupling capacitance $C_C$, as shown in fig. 2c. As the primary EQS-HBC user’s body stays at a constant EQS potential at a given point in time, this inter-body capacitance $C_C$ can couple part of that potential to a second person’s body, and can potentially be picked up by an EQS-HBC device being used by the same person. This inter-body coupling can affect EQS-HBC in two different ways, namely security and interference:

#### **Security perspective:** New attack modality with the human body as a capacitor plate

As we already mentioned, physical security of EQS-HBC has been demonstrated^2^ using E-field probes or standard RF antennas to pick up signal leakage from an HBC user. However, these probes and antennas are inefficient at the low frequency range of EQS HBC. For example, for an operating frequency of 100 kHz, an efficient mono-pole antenna will have to have a length of 750 m, which is completely impractical. However, in these low frequency range, these ’antennas’ can also pick up signal by capacitively coupling to the body of an EQS-HBC user. Now, typical electrical antennas tend to have a very small surface area, thus forming an inefficient capacitive coupling. Ideally, an electrode with a huge surface area should be able form a much better capacitive coupling with the body of an EQS-HBC user, and one of the *easiest movable semi-floating large surface area* available to an attacker is his or her own body itself. Fig. 1b, illustrates a probable attack scenario where a naive attacker with an antenna placed more than 15 cm away from an EQS-HBC user is unable to snoop the signal, whereas an informed attacker with an EQS-HBC receiver successfully does the same by using her body as a capacitive coupler and staying at a longer distance—as long as the coupling is strong enough to provide enough signal at the snooping device—and thus potentially breaks the physical security of EQS-HBC using this novel ‘Inter-Body Attack’.

#### **Interference perspective:** Proximity between multiple EQS-HBC users and impact on SIR

Inter-body capacitive coupling for EQS-HBC also poses the problem of interference between multiple HBC users in close proximity, where the signal from one user’s body can interfere with that on the other user’s body. As illustrated in Fig. 1a, for *N* number of additional EQS-HBC users with the *i*th person at a distance $d_i$ from the user under consideration, the signal to interference ratio (SIR) at that user’s body will be given by:

2 $$\begin{aligned} SIR = \frac{V_{Sig_{user}}}{V_{intf}}=\frac{V_{Sig_{user}}}{\sum _{i=1}^N V_{Sig_i}\times Coupling\left( d_i\right) } \end{aligned}$$

where $Coupling\left( d_i\right)$ is the inter-body coupling coefficient between the user under consideration, and *i*th interfering person. This coefficient is the additional loss introduced in the EQS-HBC channel path due to the physical separation of two human bodies and would be equal to 1 if the two bodies were one and the same. Functional form of $Coupling(d_i)$ is derived later in the paper as Eq. (6). For a given signal level $V_{Sig_{user}}$ on the desired user’s body, Eq. (2) should be used to determine how many other EQS-HBC users (quantified by *N*) utilizing the same frequency band could be tolerated in close proximity to that user. The number *N* in this case is to be determined based on specific use cases, e.g. number of EQS-HBC users inside an elevator, number of EQS-HBC users in a conference room, number of users within a fixed radius of someone etc.

## Results

We have motivated the fact that the coupling between two bodies in the EQS regime is dictated by the inter-body coupling capacitance, $C_C$. In the following sections, we provide a detailed account of EQS inter-body coupling, starting with a biophysical model of EQS-HBC and extending the same to incorporate capacitive coupling between two human bodies. Further, to understand the continuity from EQS to EM and the boundaries of EQS operation, we discuss forms of coupling other than EQS as well—over different frequency ranges going up to 1 GHz - where these devices behave as radiative devices instead. The theory and hypotheses described in the following section has been developed in tandem with rigorous simulations and measurements that we will discuss separately in later parts of the paper for better readability. Finally, utilizing this newfound understanding, we will propose EQS-HBC device design strategies to minimize the security and interference risks of EQS inter-body coupling.

### Different frequency regions of inter-body coupling

#### **Region 1:** Electro-quasistatic coupling

**Figure 3:**
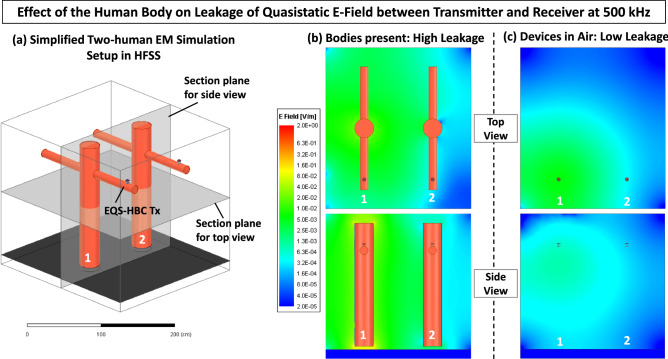
(**a**) Simulation model used in ANSYS HFSS to demonstrate leakage of quasistatic E field through the surface of the human body. The EQS-HBC transmitter on subject 1 is operated at 500 kHz, with a voltage amplitude of 1V. (**b**) Leakage of E field when the bodies are present. High surface area of the body causes a higher leakage between the bodies. (**c**) Leakage of E field in the absence of the bodies. The leaked E field is much more confined and localised around the transmitter.

**Figure 4:**
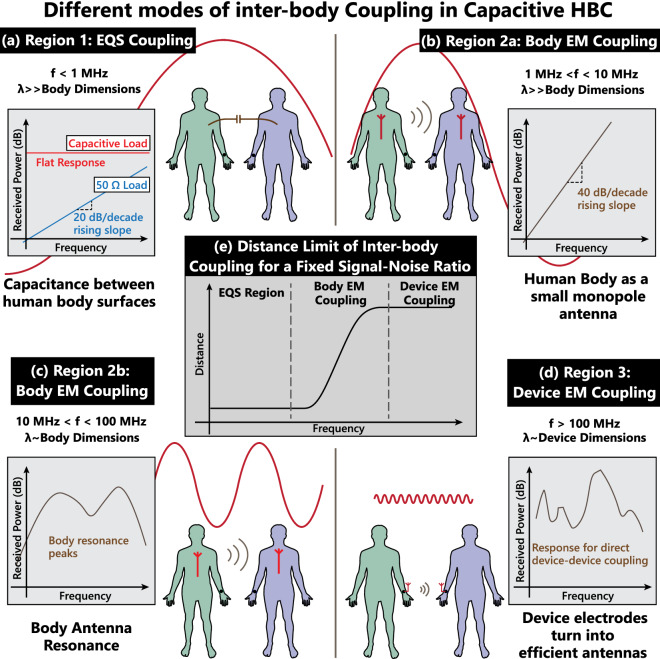
Inter-body coupling modes for Capacitive HBC users: (**a**) EQS Region, $\hbox {f}<1 \,\hbox {MHz}$, capacitive coupling dominates. (**b**) $1 \,\hbox {MHz}<\hbox {f}<10 \,\hbox {MHz}$, body starts to act as a small monopole antenna, giving a 40dB/decade rising response in coupling. (**c**) $10 \,\hbox {MHz}<\hbox {f}<100 \,\hbox {MHz}$, wavelength comparable to body dimensions; Body-antenna resonance peaks occur. (**d**) $\hbox {f}>100 \,\hbox {MHz}$, wavelength comparable to device dimensions; the devices start coupling through EM leakage. (**e**) The trend of maximum distance, over which signals from inter-body coupling can be detected (for a fixed SNR at the transmitter), over frequency. The distance limit is low independent of frequency for EQS coupling, increases rapidly once the two bodies start becoming efficient antennas, and becomes saturates once the devices become efficient antennas themselves.

This region applies to frequencies less than 1 MHz, where human body dimensions are small compared to the wavelength (Fig. 4a). As mentioned before, compared to many commercial antenna designs, the human body has a large surface area. Naturally, this can introduce a capacitive coupling between two human subjects present close to each other. In fact, this phenomenon can be demonstrated through a simple EM simulation in ANSYS HFSS—using a simplified crossed-cylinder model with dielectric and conductive properties of muscle and skin (Fig. 3a) to represent a human subject. When an EQS-HBC transmitter operating at 500 kHz is attached to one of the subjects, the electric field leaked through the body surface can be seen in Fig. 3b. When the bodies are removed from the simulation and the EQS-HBC transmitter is left hanging in air, the leaked E field is significantly lower and localized around the transmitter (Fig. 3c). This clearly demonstrates a high leakage of quasistatic E Field due to higher surface area of the body, and enables visualizing the two bodies as two ends of a capacitor. By modelling this inter-body coupling as a lumped capacitor $C_C$, and extending a simplified version of the capacitive HBC biophysical model developed by Maity et al^15^(Fig. 2d) into a two human model, a basic circuit theoretic analysis can be performed. The resulting biophysical model for inter-body coupling is presented as the circuit in Fig. 2e. There, $C_{Body}$ is the capacitance of the body surface to earth’s ground and $R_{Body}$ is the body tissue resistance. Typical experimental value of $C_{Body}$ is known to be around 150 pF^15^. $R_{Body}$ is in the order of 1 kΩ at low frequencies and its value typically reduces with increasing frequency^15,16^. The inter-body coupling capacitance $C_C$ would depend on the body surface area of the two human subjects and the distance between them—a plot of typical $C_C$ with respect to distance is shown later in the paper in Fig. 8a. As an example, for two 1.8 m tall humans standing 1 m apart, $C_C$ can be estimated to be around 20 pF. Two distinct cases of the EQS coupling region are of interest, depending on the load impedance $Z_L$ used at the receiver side—a low resistance load, typically $50\,\Omega$, and a capacitive load.

- **Resistive Load: **($Z_L=R_L$**)** For many standard RF devices, use of a $50\,\Omega$ source and load impedance is the norm. This section explores the transfer characteristics assuming a pure capacitive coupling between two human bodies. The circuit model corresponding to this case can be obtained by replacing $Z_L$ by $R_L=50\Omega$ in Fig. 2d. The coupling capacitance $C_C$ and the load resistance $R_L$ together forms a high pass filter, and the pole of the filter depends on the exact coupling capacitance $C_C$ present between two human subjects, given a fixed load resistance $R_L$. This causes a 20 dB/decade rising slope in the channel gain versus frequency plot, until at higher frequencies - where the effect of a low pass filter formed by the source resistance $R_S$ and the body shunt-capacitance, $C_{Body}$ is encountered. The resulting response from circuit simulations can be seen in Fig. 2e.
- **Capacitive load:** ($Z_L=1/\omega C_L$) For capacitive HBC, it has been suggested by Maity et al, that a capacitive load is a more viable option compared to a $50\,\Omega$ load, as it provides a flat-band frequency response in the low frequency. Now assuming the same receiver being present on a second subject, it should be interesting to see how much of the signal from the transmitting subject couples to the receiving subject. Simulating the circuit (Fig. 2d) from this modality, shows a similar flat-band response in the low-frequency region, as shown in Fig. 2e. A capacitive division is formed between the the coupling capacitance $C_C$ and the effective receiver side capacitance $C_{eff, Rx} = C_{Body}+(C_L||C_{G,Rx})$. This capacitive division is independent of frequency, giving rise to the aforementioned flat band frequency response. The inter-body channel transfer for this flat band range can be calculated to be:
  3 $$\begin{aligned} {\frac{V_o}{V_i}\approx \frac{C_{G,Tx}}{C_{Body}}\frac{C_C}{C_{Body}}\frac{C_{G,Rx}}{C_L}} \end{aligned}$$
  For frequencies above 100 MHz, a low pass effect is seen because of $R_{B}$ and $C_{tot} = C_C||C_{eff,Rx}$.

Note that the plot shown in Fig. 2f is from a circuit simulation, assuming a lumped element model of Fig. 2e. Of course, this modelling only makes sense in the EQS region ($\hbox {f} < 1 \,\hbox {MHz}$); the higher frequency regions will be explored in the following sections. In the EQS region, a capacitive load ($Z_L=1/\omega C_L$) clearly results into a consistently higher received voltage due to it’s flat frequency response—as opposed to a 20 dB/decade rising slope for the resistive load ($Z_L=50\Omega$). Further, if a regular small antenna instead of a second human body is used as a coupler at the receiver (Fig. 2b), the coupling capacitance $C_C$ would significantly drop—resulting into a much poor received voltage. In short, in the EQS region,

4 $$\begin{aligned} V_{Rx,\ Antenna\ Coupled}\ll V_{Rx,\ Body\ Coupled,\ R_L} \ll V_{Rx,\ Body\ Coupled,\ C_L} \end{aligned}$$

So if an attacker wants to device a strategy to snoop on an EQS-HBC device, the most effective strategy for them would be to use human body coupling, with an EQS-HBC receiver with a capacitive load. Hence, this is the attack modality that we will consider while suggesting design considerations for preventing snooping and interference. But prior to that, let us also briefly explore the higher frequency regions - to form an intuition about the evolution of inter-body coupling over a broader frequency range.

#### **Region 2:** Inter-body electromagnetic coupling

**Figure 5:**
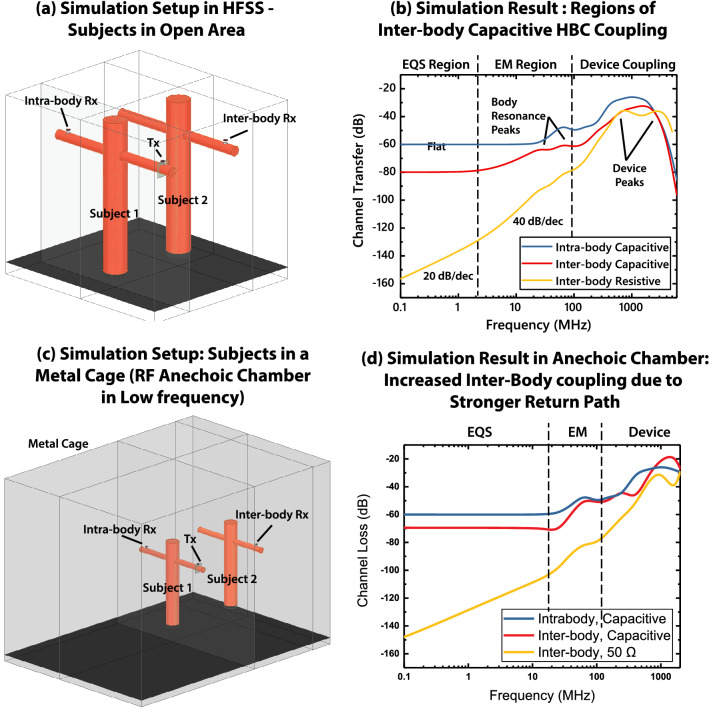
(**a**) Simulation setup used in ANSYS HFSS, using simplified models for the human body, and single-ended/ capacitive HBC electrodes as transmitter and receivers. Setup represents measurements in open-area. (**b**) Results from open-area simulation in HFSS. Three distinct regions are clearly visible, electro-quasistatic (EQS) region for freq$<1 \,\hbox {MHz}$, EM region for freq 1 MHz–100 MHz and device coupling region for freq$>100 \,\hbox {MHz}$. (**c**) Simulation setup for results in Anechoic Chamber. The subjects are enclosed in a metal-cage to represent higher return-path coupling in EQS region. This simulation setup is used to validate experimental results from EQS region in the anechoic chamber. (**d**) HFSS simulation results, inside anechoic chamber. The EQS region of the inter-body responses, are 10 dB higher compared to open air simulation results in 5b. Because of this, the transition point between EQS region and EM region moves right.

Since the human body is made with conductive tissues, it is possible to look at a standing human subject as a cylinder, made with a weak conductor. As shown in fig. 4b, a human subject standing on the earth’s ground can be seen as a monopole antenna. That being said, it should also be noted that unlike an antenna—where signal is measured between the antenna conductor and earth’s ground—in HBC, signal is measured between the body and a small floating ground. So concepts of antenna transmission may not directly apply to inter-body coupling in this case. Parts of the concepts presented in this section were developed through FEM simulations in ANSYS HFSS, discussed in detail in a later section. Based on the simulation results of HBC inter-body coupling in Fig. 5b, we will sub-divide the inter-body EM coupling region into two sections, as described below:

- *Region 2a: Electrically small monopole: * At low frequencies ($f<10 MHz$), wavelength $\lambda$ of the incident wave is large compared to the height *h* of the subject (Fig. 4b). For that reason, the body can be thought of as an electrically small monopole at these frequencies. Now the radiation resistance of an electrically small monopole antenna of length *l* is given by^4^,
  5 $$\begin{aligned} R_{rad}=80\pi ^2(l/\lambda )^2 \end{aligned}$$
  Such that, the gain of the antenna is proportional to square of the frequency—$G_{Rx}\propto f^2$. So when the received power by the body is plotted in dB vs frequency, we should see a 20 dB/decade positive slope. This behavior will be apparent in the EM simulation results discussed in the following sections. Note that when we will look at the coupling between two human subjects (Fig. 5), one of the human bodies will act as a transmitting “antenna”, while the second as a receiving “antenna”. So, the net gain at the receiver will be proportional to $f^4$, giving rise to a 40 dB/decade slope in the gain vs frequency plot.
- *Region 2b: Body resonance peaks:* For $10\hbox {MHz}<\hbox {f}<100\hbox {MHz}$, the body dimensions become comparable to wavelength. As a result, antenna resonance peaks occur, as represented in Fig. 4c. The exact position and nature of the peaks will depend on the height and posture of the subjects. A detailed analysis of the position and nature of the peaks in this region would be interesting - and while out of scope for the current paper, will be part of our future work.

#### **Region 3:** Electromagnetic coupling between devices/electrodes

The electrodes of an HBC device that is used to couple HBC signal to a subject’s body, are typically watch shaped, with a diameter of 3–5 cm. At frequencies $> 100 \,\hbox {MHz}$, these electrodes start becoming efficient antennas themselves (Fig. 4d)—peaking in the GHz range - depending on exact dimensions. As an example, if an electrode of diameter 5 cm is approximated as a mono-pole antenna of the same length, the resonant peak of the antenna occurs at 1.5 GHz in the air. In this region, the “human” part of HBC remains no longer relevant, and the communication becomes a weak form of regular wireless transmission.

#### **Summary:** Trend of maximum distance for inter-body coupling

**Figure 6:**
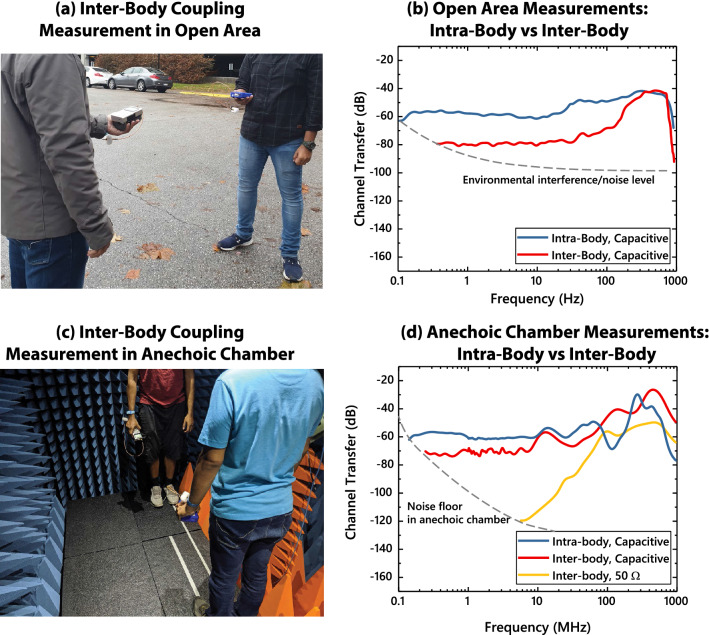
(**a**) Measurement setup in open-area. The subjects are kept at a distance of 1m for frequency sweep measurements. (**b**) Experiment results from open-area measurements. Multiple transmitting devices are used to cover the whole frequency range, as shown later in Fig. 9a. (**c**) Measurement setup inside anechoic chamber. (**d**) Results from measurements inside the anechoic chamber.

A limit of inter-body coupling distance can be conceptualized for a minimum signal to noise ratio (SNR) at the receiver for its functionality. If we focus on the capacitive HBC devices, a trend of this distance limit can be drawn (Fig. 4e) for a fixed signal level on the transmitting subject’s body. For EQS Region, since the frequency response is flat, the limiting distance is independent over frequency. As the body starts to become an efficient antenna, the limiting distance rises. Finally, when the devices themselves become efficient antenna, the limiting distance becomes flat again. The EQS region has the lowest inter-body coupling distance limit, in all three regions. In other words, given a choice of operating frequency, EQS frequency region should offer the most security against inter body coupling. This will become apparent from our simulation (Fig. 5b) and experiment (Fig. 6b) results in the following sections, where the gap between on-body or intra-body signal, and inter-body coupled signal is found to be maximum in the EQS region, and reduces in the higher frequency regions.

### Results from FEM simulations and experiments

So far, we have discussed the different modalities through which signal transfer could happen between two human subjects wearing an HBC transmitter and receiver respectively. In a real-world scenario, all these effects are present simultaneously, and depending on the region in the frequency spectrum, one of these can become dominant. We show results from EM simulations as well as experiments in this section to demonstrate this very fact. For simulations, ANSYS HFSS—an FEM based Maxwell’s equations solver is used. A simplified human body structure is assumed as shown in fig. 5a. Additional details about both the EM simulation setup and experiment setup can be found later in the Methods section.

#### HFSS simulation for frequency dependent inter-body coupling transfer characteristics

Two subjects are kept at a distance of 1m from each other, with capacitive HBC device models on stretched arms. Simulation is performed over the frequency range of 100 kHz–1 GHz, for both capacitive and $50\Omega$ termination at the receiving subject’s device. The resulting transfer characteristics is shown in Fig. 5b. Evidently this transfer characteristics can broadly be divided into three regions, depending on the dominant modality of coupling in operation:

- *Freq* < *1 MHz*: In this region, we see a 20dB/decade rising slope for the $50\,\Omega$ termination, and a flat band response for the capacitive termination. This indicates that the dominant coupling method in this frequency range is electro-quasistatic, and hence can be modeled by circuit models shown in Fig. 2c
- *Freq 1 MHz–100 MHz*: In this region for the $50\,\Omega$ termination, we see a 40 dB/decade rising slope that flattens into peaks between 20 and 80 MHz. This indicates electromagnetic/ mono-pole antenna coupling between the two subjects. For capacitive termination, we see an increased response from the flat-band in the lower frequency range and peaks at similar frequencies as the $50\,\Omega$ termination. The slope is less than 40 dB/decade however - this indicates that both EQS and EM effects are equally present in this case—the EQS effect being a flat-band response at − 80 dB, while the EM effect being a 40 dB/decade rising slope. When the two effects are added, a gentler rising slope results - and the peaks from the EM effect show up at a higher level (lower loss) compared to the $50\,\Omega$ termination.
- *Freq* > *100 MHz*: In this frequency range, we see a sharp rise in the transfer characteristics, due to the electrodes becoming efficient antennas. This becomes the dominant mode of transfer, as in the GHz frequency range, the human body becomes an inefficient antenna. Its resonant frequency as a mono-pole antenna lies in the 20–80 MHz range, and the response starts dropping as the frequency is increased beyond that, as shown in Fig. 5b. Transfer of signal by EQS capacitive coupling between the two subjects also becomes inefficient due to the previously discussed low-pass effect (Fig. 2e). So in the high frequency range ($>100 \,\hbox {MHz}$), a sniffing device that has a relatively small form-factor, like a hand-held antenna, can pick up leakage signal from an HBC device efficiently. Also note that the difference between intra-body and inter-body signal levels in this region is much lower compared to the flat  10-20 dB difference in the EQS region of $f<1MHz$. This pretty much renders this frequency region unsafe for operating HBC devices, reiterating the importance of EQS region in HBC—and the focus of the current paper.

#### Experiments

To validate the simulation results, we perform experimental channel loss measurements between two human subjects—with the first set of experiments performed inside an anechoic chamber for clean results, free from external interference. The subjects are kept at a distance of 1 m, and a frequency sweep at the transmitter is performed from 100 kHz to 960 MHz. We use handheld devices for our measurements as opposed to wall connected devices, as wall connected devices share a common ground, and would hence reduce the channel loss and produce an inaccurate result. Now to cover the entire frequency range of our experiments, we split the range into multiple handheld RF generators—details of which can be found in the Methods section. Note that the ground sizes of the different transmitting devices are slightly different, and that in turn makes the transmitter side return path capacitance, $C_{G,Tx}$ slightly different between the devices. As a result, there are slight discontinuities in the plot of the measurement data, in Fig. 6b and 6d. However, we made sure to minimize these discontinuities through a rigorous calibration protocol, described later in the methods section.

While performing the experiments inside the anechoic chamber (Fig. 6c) provides a controlled low-noise environment for gathering accurate frequency response, the chamber is enclosed in a grounded metal cage and that affects the low frequency EQS region of the results. First, the grounded metal cage increases the overall return path capacitance, and that reduces channel loss. Second, as the EQS region now shows lower loss, the crossover point between the EQS and EM regions moves to a higher frequency. The chamber used for our experiments is rated to efficiently absorb incident EM waves above 80 MHz; so the results from anechoic chamber can be correlated with the HFSS open air simulations only above 80 MHz. To reproduce the anechoic chamber conditions in the low frequency EQS range, a second set of simulations are performed where the anechoic chamber is modelled as a metal cage (Fig. 5c). The results from that simulation—shown in Fig. 5d—show an improved correspondence in the EQS range with experiment results in anechoic chamber (Fig. 6d). Anechoic chamber measurements (Fig. 6d) show a 10 dB reduction in inter-body coupling loss in the EQS region compared to open-air simulation results in Fig. 5b. Also, the cross-over point between EM and EQS region moves close to 10 MHz, as opposed to 1 MHz in Fig. 5b.

The experimental measurements are also repeated in an open area (Fig. 6a) to eliminate the effect of a metal enclosure in the results. Environmental RF noise presents a challenge in open area measurements however - especially for the $50\,\Omega$ receiver load case in the low frequency region. We present an averaged data for the capacitive load cases over multiple measurements in Fig. 6b. The measurement data reproduces the 20 dB difference between intra-body and inter-body signal level in the flat EQS region, as seen earlier in simulations (Fig. 5b).

From our earlier example in the anechoic chamber, we saw that intra-body and inter-body losses are 60 dB and 70 dB respectively. Since the anechoic chamber provides a strong return path, this was an optimistic estimate for inter-body channel loss. On the other hand, a pessimistic estimate of the channel loss comes from the open-air case, where the inter-body loss is about 80 dB. So even at 1 m distance between the two bodies, the difference between intra-body and inter-body channel loss lies between 10 and 20 dB.

### FCC regulations: Can EQS-HBC device be classified as an unintentional radiator?

In the previous section we have looked into the inter-body coupling among humans when they are using EQS-HBC as BAN communication. This coupling depends on the Electric fields created by the EQS HBC User and the surface area of the recipient. Related to the phenomena of electric fields around the human body during EQS HBC transmission, an important question arises about the usability of these devices in practice: Can EQS-HBC Device be classified as an Unintentional Radiator?

**Figure 7:**
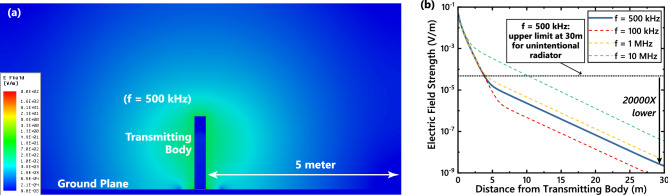
(**a**) Electric field decay from an EQS HBC device in a 2-D cross section at 500 kHz. (**b**) Plot of E-field decay vs distance shows that the E-Field drops 20000x below the FCC threshold to qualify as an unintentional radiator. This seconds the weak capacitive coupling demonstrated in Fig. 2b.

According to FCC regulations^17^ as shown in Table 1, the definition of intentional vs unintentional radiator is as follows: for a frequency F between 9 and 490 kHz, if the fields at 300 m distance are below 2400/F and for a for a frequency F between 490 kHz and 1.705 MHz, if the fields at 30 m distance are below 24000/F, the device can be classified as an unintentional radiator—which means no additional FCC certification would be required for deployment of these devices in practice. Using our developed model, we can get a great sense of the “radiated” electric fields in EQS-HBC. In Fig. 7a, electric field emission from a human body with an active EQS-HBC transmitter is visualized. From the decay of the field vs distance plotted in Fig. 7b, it can be seen that at 30 m distance, the electric field is about 20000 times lower than the required FCC limit (Table 1). So, the fields emanated from the EQS-HBC devices are low—and is not perceptible by other devices as per FCC standards. Hence EQS-HBC devices can be classified as unintentional radiators, and can be deployed without the need for new standards and certifications.

**Table 1:** FCC field limit regulations for unintentional radiators^17^.

Frequency (MHz)	Field Strength ($\mu \hbox {V/m}$)	Measurement Distance (m)
0.009–0.490	2400/F (kHz)	300
0.490–1.705	24000/F (kHz)	30
1.705–30.0	30	30
30–88	100	3
88–216	150	3
216–960	200	3
Above 960	500	3

### EQS-HBC design consideration to maximally protect against inter-body attack and interference

**Figure 8:**
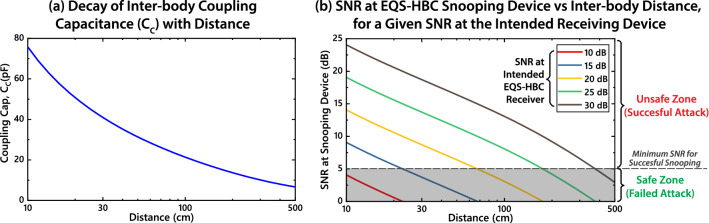
(**a**) Inter-body coupling capacitance ($C_C$) with distance, obtained from simulating the model shown in Fig. 5a with varying inter-body distances in ANSYS Maxwell. (**b**) SNR at a snooping device, for a given SNR at the intended receiving device. When the SNR at the snooping device falls below 6–9 dB, successful attack is prevented.

We have shown the different regimes of inter-body coupling in HBC. More specifically, for EQS-HBC ($\hbox {f}<1 \,\hbox {MHz}$), inter- body transfer characteristics show a flat band response similar to intra-body HBC, given a capacitive load is used at the receiving device. The difference in the channel loss between these two cases determines whether a successful attack can be performed using the human body as a capacitive coupler. By comparing the equations shown in Fig. 2d,e, the difference in channel loss between these two cases—or the coupling coefficient *Coupling*(*d*) from Eq. (2)—can be given by:

6 $$\begin{aligned} Coupling(d)=\frac{V_{Inter-body}(d)}{V_{Intra-body}}=\frac{C_C(d)}{C_{Body}} \end{aligned}$$

where *d* is the distance between the EQS-HBC user and the attacker’s body. Since the body to ground capacitance $C_{Body}$ is fixed at around 150 pF^15^, variation of the inter-body coupling capacitance $C_C$ with *d* will determine the variation of $V_{Inter-body}$ with *d*. Figure 8a shows a plot of $C_C$ vs *d*, obtained by electrostatic FEM simulation in ANSYS Maxwell. Accordingly, putting $C_C=21 pF$ for $d=1$ m in Eq. (6), we find an additional loss of  17 dB for inter-body coupling. This matches with our previous experimental finding, where we saw that at 1 m distance, the difference between intra-body and inter-body channel loss was in the range of 10–20 dB. At 5 m, $C_C$ reduces to 6.6 pF, which raises this difference to 27 dB.

**Table 2:** Minimum SNR required for operating at three different intended uncoded bit error rate (BER) in the cases of PAM 2, QPSK and 16-QAM modulation techniques^18^.

Intended BER	Minimum SNR (dB)		
	PAM 2 (OOK)	QPSK	16-QAM
$10^{-2}$	4.3	1.2	9.6
$10^{-3}$	6.8	5.3	11.9
$10^{-4}$	8.4	7.1	13.2

Now, let us consider the example of EQS-HBC using On-Off Keying (OOK) signals^19,20^, also known as PAM-2 modulation and target a coded bit error rate (BER) in the range of $10^{-6}$–$10^{-8}$. This would imply an uncoded BER of about $10^{-3}$ and set the minimum signal to noise ratio (SNR) requirement at about 6–7 dB^18^. So to ensure proper functionality of intra-body EQS-HBC communication with this specific modulation and BER criteria, transmitter power should be kept 6–9 dB greater than the receiver sensitivity including intra-body channel loss. We can then calculate the additional loss for inter-body coupling using Eq. (6) and $C_C$ from Fig. 8a. This enables us to estimate the SNR at the snooping person’s receiver for any pre-determined SNR at the intended receiver on the EQS-HBC user’s body. This is plotted in Fig. 8b for a set of given SNR at the intended receiver. The shaded “safe zone” is set below an SNR of 5 dB, due to the minimum 6–7 dB SNR requirement for this specific example. By staying in the shaded region in the plot, a successful attack can be prevented. For example, if the signal level of the EQS-HBC transmitter is set to maintain an SNR of 10 dB at the intended receiver, an attacker will not be able to snoop that signal even at 10 cm distance from the user.

This approach of setting the transmitter signal level can easily be generalized for any modulation scheme and targeted BER, by referencing the corresponding BER/SNR data. Table 2 lists SNR requirements for a couple of frequently used modulation schemes for different targeted uncoded BER. By modifying the upper limit of the “safe zone” in Fig. 8b according to the minimum SNR requirement from Table 2 or otherwise, a designer can make an informed choice regarding the signal level of an EQS-HBC transmitter. Further, by setting the signal level in this way, interference effects are also reduced between multiple adjacent EQS-HBC users in a common space. So even if inter-body coupling in EQS-HBC introduces a risk of unintended signal sniffing and/or interference, steps can be taken towards setting the signal level of an HBC device to minimize or eliminate the possibility of the same.

Additionally, we would like to comment that the design method proposed above is meant to be used for determining an optimal *static power* of an EQS-HBC system. A static power based EQS-HBC system is viable because unlike wireless systems, channel variability in EQS-HBC is primarily dependent on the transmitter and receiver sizes and much less on specific on-body locations of the transmitter and the receiver^14^. This in turn conveniently makes the communication safe from a pulsed interference attack, that could have potentially stolen data from an adaptive power based system. For a static power based system, this kind of attack would only cause jamming or denial of service, without the risk of data theft.

## Conclusion

In conclusion, we show that the human body can function as a capacitive coupler to pick up EQS-HBC signals, making this BAN technique vulnerable to inter-body attack and interference. We explore inter-body coupling modalities over a broad frequency range (100 KHz–1 GHz). We identify and explain three distinct regions—namely EQS inter-body coupling, inter-body EM coupling and inter-device EM coupling. We postulate a biophysical model that describes inter-body coupling in the EQS frequency region ($< 1 \,\hbox {MHz}$) as a function of the capacitance between two human bodies, which in turn is a function of distance. Finally, we demonstrate that by optimizing the signal level at a EQS-HBC transmitting device, the inter-body coupling vulnerabilities can be reduced (if not eliminated) to a distance of less than 10 cm of an EQS-HBC user’s body, restoring the physical security of EQS-HBC.

## Methods

This section contains details regarding our simulation and experimental methods, to facilitate reproduction of the results if anyone wishes to do so.

### EM simulation setup

All the EM simulations have been performed in Ansoft HFSS, which is a Finite Element Methods based Maxwell Equation solver. A simple crossed cylinder model is used in place of a human body for simplicity and fast simulations. A detailed model consisting of different human tissue parts is also used to validate the simple model’s accuracy. Dielectric properties of all body tissues have been taken from the works of Gabriel et al^16^.

#### Simple crossed cylinder model

A simple model is created using two perpendicular cylinders, as shown in Fig. 5a, representing the torso and extended arms. The radius of the cylinders are 14 cm and 6 cm respectively. The height of torso is taken to be 180 cm, and the entire arm span is taken to be 180 cm as well. Both the torso and the arms are divided into a 4 mm outer shell of skin, and an interior of muscle. This crossed-cylinder model is floated 2 cm above a plane with Perfect E Boundary in HFSS—supposed to replicated an infinite ground plane or the earth’s ground. A rubber cylinder of same diameter as the torso is placed between the torso and the perfect E plane. The entire model is then enclosed in a region of air, measuring 120 cm *times* 60 cm *times* 340 cm. Excitation for the simulation is provided through capacitive coupling, as described in the next sub-section.

#### Excitation

A capacitive coupling model is used to provide excitation to the body attached to a transmitter. The coupler consists of two copper discs with a radius of 2.5 cm. One of the discs, is 2 mm thick and is curved onto the arm—this disc replicated an electrode patch attached to the arm. The other disc with a thickness of 5 mm, replicates the ground plane of an wearable watch-like HBC device. the separation between the two plates can be varied to change the capacitance between the plates, a distance of 3 cm is used in our simulations, yielding an approximate parallel plate capacitance of 0.6 pF. Alternatively, a fixed capacitance of choice can be maintained between the plates, using a lumped RLC boundary in HFSS. A voltage source excitation is placed between the two plates. In HFSS, this imparts an alternating potential difference of amplitude 1 V between the two plates, replicating an ideal AC voltage source. This is unlike the lumped port excitation method in HFSS, which is ideal for $50\Omega$ matched excitations, but may give rise to unexpected reflections when coupling to a non-standard RF model.

#### Measuring voltage at receiver

The receiving node structure is almost identical to that of the transmitter, with parallel discs of similar dimensions. A lumped RLC boundary is placed between the electrode and the ground plate at the receiver, which is set to $50\Omega$ for a low impedance termination, and 1 pF for capacitive high impedance termination cases. The potential difference between the plates is calculated by integrating the electric field along a straight line between the electrode and ground plates. Note that for capacitive termination, the size and shape of the receiver ground plate controls the ratio $C_{G,Rx}/C_L$ in Eq. (3). To maintain a fast simulation time, we refrained from modelling the exact experimental receiver in detail and stayed with the simpler parallel disc structure. This however makes both $C_{G,Rx}$ and $C_L$ different from that in the experimental setup and we found that artificially setting $C_L = 1$ pF in the simulation restores the $C_{G,Rx}/C_L$ ratio to yield results comparable to the experiments. Increasing or decreasing the value of $C_L$ would result into a decrease and increase respectively of the level of the EQS flat-band in Fig. 5b,d.

#### Calculating inter-body coupling capacitance ($C_C$)

The distance dependent inter-body coupling capacitance $C_C(d)$ is calculated by simulations in ANSYS Maxwell, which is an FEM based static Maxwell’s Equations solver. Simulations are performed in Maxwell’s electrostatic mode. The same two person cross-cylinder model as shown in Fig. 5a is used here as well, and the capacitance matrix between the two bodies is calculated by assuming the individual bodies as individual conducting objects. The distance between the two bodies is varied to obtain $C_C$ as a function of *d*, and the resulting plot is shown in Fig. 8a.

#### The case for a detailed model

As the reader might have noted, all the EM simulations presented in this paper has been performed with a simplified crossed cylinder model of the human body that only includes skin and muscle. This may appear as an oversimplification. However, we did validate the simple model by comparing the fields and currents in and around the model to that of a more detailed model, specifically VHP Female v2.2 from Neva Electromagnetics^21^. In fact, a previous work by Maity et al^22^ performed this comparison in detail and showed that the field distributions inside and outside the model and received signals are similar between the simple and complex model cases. Intuitively this makes sense when we look at the electrical properties of different tissues on the body^16^—dielectric permittivity of most human tissues (except blood) are orders of magnitude higher than that of the air, and most of them exhibit a low yet non-negligible conductivity. So when contrasted with air that has a relative permittivity of 1 and zero conductivity, the body largely behaves like a homogeneous mass of high dielectric permittivity and low but positive conductivity—in the context of electromagnetic fields. And since HBC devices discussed in this paper operate at the interface of air and the body, the same simplification holds true. This makes the simple crossed cylinder model yield meaningful results without any loss of generality and reduces computational complexity and time by orders of magnitude—letting us perform simulations over multiple frequencies and configurations.

### Experimental setup

Experiments are conducted in two parts—the first set of measurements are made inside an EM anechoic chamber to maintain a controlled environment and achieve noise immunity. The second sets of experiment are done in an open area such as an empty parking lot, to compare signal levels with the ones inside anechoic chamber. For the purpose of replicating real-world HBC devices, hand-held transmitting and receiving devices are used, as opposed to wall connected such as a Vector Network Analyzer. Wall connected devices essentially share a common ground and bring the ground planes of the transmitter and receiver at a common potential, thus showing a lower loss and giving an optimistic channel transfer characteristics^14,15^.

**Figure 9:**
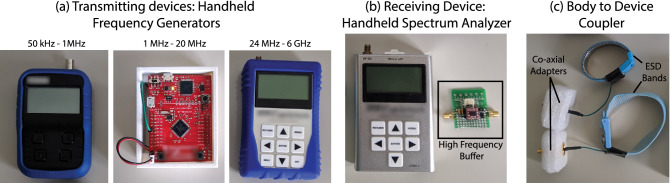
Devices used for experiments: (**a**) Transmitting devices: Velleman Handheld RF Generator for freq$<1 \,\hbox {MHz}$, signal generator built using a Tiva C Launchpad Board for freq 1MHz–20MHz and RF Explorer Handheld RF generator for freq$>24 \,\hbox {MHz}$. (**b**) RF Explorer Handheld spectrum analyzer used as a receiving device, and a high-frequency buffer used for high impedance/capacitive load measurements. (**c**) Couplers used to connect the transmitter and receiver devices to body.

#### Transmitting devices

We plot the transfer characteristics over a large frequency range, namely   100kHz–1GHz. We use multiple handheld RF signal generators (Fig. 9a) to cover the entire range:

- *Freq* < *1 MHz* A hand-held signal generator from Velleman is used to generate sinusoidal signal for frequencies lower than 1 MHz.
- *Freq 1 MHz–20 MHz* An in-house signal generator is used, built using a Texas Instruments Tiva C Launchpad evaluation board. The generator provides a square wave, the fundamental harmonic is used for our experiments.
- *Freq* > *24 MHz * RF explorer handheld RF generator is used, which generates sine wave in the range 24 MHz–6 GHz

All the transmitting devices are characterized using a precision spectrum analyzer, to record accurate transmitting power of the fundamental at each frequency point. The power of the transmitting devices are controlled such that the peak-to-peak voltage applied to the body stays around 2–4 V. As shown previously by Maity et al^22^, this ensures that the fields and current densities inside the body stay well within the limits of safety mandated by ICNIRP standards^23^. The experimental protocols involving human subjects have been approved by the Purdue Institutional Review Board (IRB Protocol $\#$1610018370). All guidelines and regulations, as given by the Purdue IRB were followed during the experiments. Informed consent was obtained from all the participants for the experiments.

#### Receiving devices

We use a handheld spectrum analyzer from RF Explorer that covers 50 kHz–960 MHz. The range is adjusted for each frequency point measurement, to include only the fundamental peak, and the peak power is noted. Subtracting the characterized transmitter power from this received power provides the channel transfer gain at that frequency. For measuring the $50\,\Omega$ termination cases, the spectrum analyzer is directly connected to the HBC coupler, as the device has an input impedance of $50\,\Omega$. For capacitive load measurements, a high-frequency buffer is connected to the HBC coupler first, and the buffer’s output is given to the spectrum analyzer. The buffer board, shown in Fig. 9b, is made using BUF602, a high speed buffer from Texas Instruments. The board is configured to have an input resistance of $1 \,\hbox {M}\Omega$. Ideally this would just make the input impedance of the receiver as $1 \,\hbox {M}\Omega$ resistive. However, in reality a parallel parasitic capacitance forms between the receiver electrode and receiver’s ground plane—which in our case is the ground plane of the buffer board and the chassis of the spectrum analyzer. This capacitance was characterized to be around 10 pF. For our frequency range of interest ($f>100\,\hbox {kHz}$), the impedance from this 10 pF capacitance is much lower compared to the default $1\, \hbox {M}\Omega$ input impedance of BUF602, and hence the net input impedance essentially becomes capacitive. If the frequencies were to be lowered from 100 kHz, this approximation would indeed stop holding at some point and a high pass effect similar to that of the $50\Omega$ termination case would be seen. The purpose of the buffer is to move this transition point to a frequency much lower than the frequency range of interest, and maintain a capacitive termination.

#### Calibration

As we have mentioned before, characterization of HBC systems demands the use of small form-factor wearable/portable transmitting and receiving devices for accurate channel measurements. Unfortunately, that prevents us from using precise bench-top measurement equipment. This calls for careful calibration of all the transmitting and receiving devices to ensure accurate measurement results.

- To calibrate the three different transmitting devices, each of them is individually connected to a precise bench-top Keysight spectrum analyzer. For each frequency point of interest, the displayed power of the fundamental peak at the spectrum analyzer is recorded. This record is used as a reference frequency dependent transmitted power for all the transmitting devices.
- The accuracy of the receiving hand-held spectrum analyzer is also examined by connecting it directly to a precise bench-top Keysight RF signal generator, and applying a sinusoidal RF signal at individual frequencies of interest. The power of the applied RF signal is kept within the order of expected on-body received power. Any deviation of the displayed power at the hand-held spectrum analyzer from the applied power is recorded to be applied as a correction to future measurements.
- Finally, the buffer is also characterized using the same Keysight signal generator and spectrum analyzer, and it’s frequency characteristic is recorded.

To calculate the channel transfer at a given frequency, the previously recorded transmitted power is subtracted from the measured received power. Correction for receiver deviation is then applied, and additionally the result is adjusted for the buffer characteristics in case of capacitive termination measurements.

#### Body to device coupler

To couple the transmitting and receiving devices to a subject’s body, an ESD wristband is used, worn on the subject’s arm. The signal pin of a co-ax adapter is connected to the metal plate of the ESD band by soldering a small piece of wire. An example of the coupler is shown in fig. 9c. These couplers are in turn connected to the transmitting and receiving devices using a shielded co-ax cable.

#### Frequency sweep measurements

To obtain leakage or inter-body transfer characteristics over frequency, the experiment subjects are asked to stand 1 m apart, facing each other. The transmitting device is coupled to one subject while the receiving device to the other. The subjects operate the handheld transmitting and receiving devices themselves to sweep frequency by hand. The receiving subject communicates the resulting peak power to a third person standing away from the two subjects, to log data.
